# Mental health challenges in Nigeria: Bridging the gap between demand and resources

**DOI:** 10.1017/gmh.2024.19

**Published:** 2024-02-16

**Authors:** Kehinde Precious Fadele, Stephen Chukwuemeka Igwe, Niji-Olawepo Toluwalogo, Ephraim Ikpongifono Udokang, Jerico B. Ogaya, Don Eliseo Lucero-Prisno

**Affiliations:** 1 College of Medicine, University of Nigeria, Enugu, Nigeria; 2 College of Health Sciences, University of Ilorin, Ilorin, Nigeria; 3 Faculty of Pharmacy, University of Uyo, Akwa Ibom State, Nigeria; 4 Department of Medical Technology, Institute of Health Sciences and Nursing, Far Eastern University-Manila, Metro Manila, Philippines; 5 Department of Global Health and Development, London School of Hygiene and Tropical Medicine, London, UK; 6 Faculty of Management and Development Studies, University of the Philippines Open University, Laguna, Philippines; 7 Faculty of Public Health, Mahidol University, Bangkok, Thailand

**Keywords:** mental health, mental well-being, psychiatrist, Nigeria, Challenges

## Abstract

This study discusses the significant topic of mental health challenges in Nigeria, focusing on the shortage of mental health professionals, particularly psychiatrists, and the factors influencing medical students’ hesitation to pursue psychiatry as a career path. We examine the multifaceted difficulties in Nigeria’s mental health environment using various sources, including research studies and statistical data. The scarcity of psychiatrists, with only 250 for a population of 200 million, underscores the urgent need for attention to mental health within the country. Factors such as brain drain, inadequate learning infrastructure, limited research exposure and insufficient health coverage contribute to this crisis. Additionally, societal stigma and financial constraints discourage students from pursuing psychiatry as a career. To address these challenges comprehensively, we propose a holistic approach that involves cross-disciplinary collaboration, robust mental health education in all healthcare programs, community-based awareness initiatives and transdisciplinary teamwork among mental health providers. We highlight the importance of mentorship, scholarships and national advocacy to encourage more individuals to enter the mental health profession, emphasizing the need for diversity and inclusiveness. Furthermore, our paper stresses the significance of research and innovation in advancing mental health treatment and inspiring passion for mental health among students and aspiring professionals. By embracing this comprehensive set of recommendations, we aim to cultivate a diverse, talented and compassionate workforce capable of effectively addressing Nigeria’s pressing mental health challenges.

## Impact statement

It is no news that mental health challenges and illnesses are on the rise in present times. The socioeconomic and political climate and other biopsychosocial factors have been implicated as factors contributing to the fast-rising burden of mental health. While other parts of the world are catching on to this realization and making an effort to match the demand for mental health care with the supply of caregivers and professionals, Africa and Nigeria in particular still have a long way to go. From trained psychiatrists and psychologists to paramedics and other personalities in mental health, there is a dire need to draw people into the field of mental health and psychiatry. Our paper aims to identify the challenges that plague the wide gap between the demand and supply of health care in Nigeria. From the exposure of medical students to psychiatry to issues like lack of adequate research and health coverage, we break down the factors that contribute to the deficiency of mental health care in Nigeria. We then go on to outline recommendations that can cause positive changes in this detrimental trend. One of our major recommendations is that from medical students and psychiatrists, it is important for students in other departments as well as members of the public to become involved in mental health. We emphasize why it is important for all young people in all facets, and works of life to become mental health advocates and direct or indirect collaborators in the delivery of mental health care in Nigeria.

## Introduction

Across the globe, there is a growing recognition of the significance of mental health and the field of psychiatry within medical specialties. According to the World Health Organization (2017), approximately 450 million people grapple with mental illnesses, and an estimated 25% of the world’s population will encounter mental health challenges at some point. Furthermore, mental disorders contribute to approximately 7% of the global health burden, impacting nearly 19% of individuals with disabilities (James et al., 2018; Rehm and Shield, 2019). This heightened awareness has led to widespread initiatives, encompassing awareness campaigns, advocacy rallies, the establishment of nongovernmental organizations, the expansion of hospital facilities, the dedication of passionate professionals and governmental interventions, all aimed at fortifying the support system for mental health and psychiatry. However, while many regions have embraced this progress, certain parts of the world lag, with Nigeria notably standing at the forefront of this challenge.

Numerous factors underlie the inadequacy of mental health care services in Nigeria, with one of the most prominent being the shortage of psychiatrists within the country. Astonishingly, despite a population of 200 million people, Nigeria boasts only 250 practicing psychiatrists (Association of Psychiatrists of Nigeria, 2018). This glaring disparity in the Psychiatrist-to-population ratio underscores the marginalization of mental health within the country, a situation exacerbated by the issue of brain drain, which continues to deplete the healthcare workforce in Nigeria (Esan et al., 2014).

However, the scope of addressing mental health extends beyond psychiatrists alone. A multifaceted approach necessitates the involvement of various healthcare professionals, including social workers, psychologists, community health experts, occupational therapists, mental health pharmacists, counselors and community support workers. The factors influencing mental health are wide-ranging, encompassing familial, social, economic, environmental and more (World Health Organization, 2022). Therefore, effective mental health care and management demand a holistic approach extending beyond the confines of hospital walls. In contemporary Nigeria, addressing mental health challenges requires a collective effort, engaging individuals and platforms at the grassroots level, with interventions spanning sectors such as education, transportation, welfare, housing and beyond (World Health Organization, 2022).

Amid the mounting burden of mental illnesses in Nigeria, a pressing need arises to delve into the factors that shape public perceptions and participation in mental health initiatives. An essential facet of this exploration is identifying elements influencing medical students’ choice of specialization after completing their medical education. Understanding these influences is pivotal in elucidating the significance of psychiatry within the medical field and, in turn, encouraging more medical students to consider psychiatry a viable career path.

## Mental health challenges in Nigeria

Nigeria today confronts a state of emergency in mental health. Based on Ugochukwu et al. (2020), patients with psychiatric care needs are often rendered to their household members since the available mental health workforce is apparently out of proportion, the majority of whom are centralized in urban areas, compounded by the lack of knowledge and capacity to cater mental disorders at the primary healthcare level (Ugochukwu et al., 2020). Statistics indicate that about 80% of people in Nigeria with severe mental health needs are unable to obtain care, which is primarily attributable to the country’s stigma and negative social attitudes toward mental health issues as well as a lack of facilities, resources and mental health professionals (Demyttenaere et al., 2004). It is of utmost importance to recognize the unique socioeconomic and cultural barriers that can impede mental health outcomes in Nigeria and tailor interventions to meet the needs of local communities.

### Inadequate learning infrastructure

The Nigerian medical school system faces challenges in utilizing emerging instructional tools, such as mannequins and simulations, leading to a reliance on imagination in teaching psychiatry and hindering practical learning and comprehension (Bzdok and Meyer-Lindenberg, 2018). The lack of effective training in mental health services is also evident in the pharmacy curriculum, where inadequate knowledge hinders the provision of mental health services by pharmacists (Bamgboye et al., 2021). Furthermore, the implementation of e-learning in Nigerian schools is primarily limited to traditional methods, such as lecture notes and prerecorded audiotapes, highlighting the need for more advanced instructional tools (Oriji et al., 2023). These issues call for a comprehensive reform in the medical education system to address the gaps in mental health education in Nigeria.

### Inadequate research exposure

Nigeria incorporates practical research findings from industrialized nations to enhance its mental health services. While some of these findings have aided in improving healthcare, others have fallen short, as they cannot be extrapolated due to a lack of relevance to the unique needs and challenges of the country. In the academe, students are being introduced and supervised to conduct research projects for the first time during their last year of study in most Nigerian medical schools as part of the mandatory graduation requirements. Since most students view these projects as mere compliance to graduate rather than an essential component of medical education, many students fail to get the necessary knowledge. They cannot develop research skills in such a short period. This invariably results in physicians with poor research abilities and hinders the growth of medicine in the country (Osoba et al., 2021).

### Lack of health coverage

According to pertinent measures, although Nigeria has improved health insurance, its overall health coverage remains inadequate and unequal, with far-reaching social disparities between the rich and the poor and between those who reside in rural and urban areas (Chukwudozie, 2015). On top of the inadequate and disproportionate healthcare service delivery in Nigeria, with the limited availability of mental health professionals compared to other African and low-income countries, providing better coverage of mental health treatment becomes an uphill battle. However, properly integrating mental health services into primary care using the task-sharing principle, a strategy successfully piloted in Nigeria, may be a practical approach to increase accessibility to mental health care services (Abdulmalik et al., 2019).

### Utilization of mental health services

One of the significant difficulties in managing and controlling mental disorders is the utilization of inadequate mental health services. WHO deemed it a critical factor as there has been a claim that most people with severe mental and neurological issues are those who have reluctant behavior toward utilizing mental health facilities – more than 80% in developing countries like Nigeria and approximately 50% in industrialized countries (Kukoyi et al., 2022).

The utilization of mental health services in Nigeria presents complex challenges, as evidenced by various studies. Research indicates that a significant proportion of individuals with mental disorders in Nigeria initially seek alternative sources of care, such as spiritualists or herbalists, before utilizing formal mental health services (Lasebikan et al., 2012). Additionally, there is a high level of unmet need for mental health services in Nigeria, with a substantial portion of individuals not receiving adequate care for their mental health conditions (Lasebikan et al., 2012). Regional variations in mental health service utilization are also evident, as demonstrated by a 10-year study in northeastern Nigeria, highlighting the need for tailored approaches to address utilization patterns in different areas of the country (Mohammed Said et al., 2015).

Furthermore, the integration of mental health into primary care in Nigeria has been explored, emphasizing the importance of leveraging the primary health care system to deliver mental health services at the community level (Abiodun, 1995; World Health Organization, 2007). This approach aims to improve access to mental health care and address the challenges associated with the underutilization of formal mental health services. Additionally, the treatment received by individuals with serious common mental disorders in Nigeria has been examined, revealing that a significant percentage of these individuals do not receive adequate treatment, indicating gaps in the utilization of mental health services (Wang et al., 2007).

## Factors hindering medical students’ interest in psychiatry in Nigeria

Several circumstances influence medical students’ career speciality decisions, which may be inferred early, during, or before their medical school training. These are divided into pre-, intra- and post-medical school factors, focusing on pre- and intra-medical school factors (Farooq et al., 2013). Gender, exposure to mental illness, the social stigma associated with mental illness and perception of psychiatry are premedical school factors related to choosing psychiatry. At the medical school level, factors influencing career options include attitudes toward psychiatry, teaching methods, the quality and length of clinical exposure, electives and enrichment activities, personality factors and career guidance.

Students who pursue to become psychiatrists later in life have some premedical school factors in common. Gender is one of these factors, and there has been a beneficial association between the female gender and a positive attitude toward psychiatry (McParland et al., 2003). Prior knowledge of mental illness before entry to medical school, either personally or through having a family member or a friend become mentally ill, was associated with a positive attitude toward psychiatry and a choice in the specialty as a career in another study conducted by Kerebih et al. (2019) among medical undergraduates in Ethiopia. This is supported by a survey conducted by Rajagopal et al. (2004), which suggests that it could be due to the student’s experience knowing or caring for a mentally ill person, which makes the student more comfortable with psychiatry and psychiatric patients. They also state that another possibility is that these students have firsthand knowledge of the flaws in psychiatric care service delivery and want to make a difference. Nevertheless, societal attitudes toward psychiatry and mental illness can influence students’ career choices.

Mental illness is often stigmatized in Nigeria, as it is in many other countries, and this stigma can discourage students from pursuing psychiatry as a career (Ighodaro et al., 2015). This is especially concerning given that Nigeria already has a severe shortage of mental health professionals, with only one psychiatrist for every 800,000 people (Association of Psychiatrists of Nigeria, 2018). Furthermore, many medical students in Nigeria face financial constraints and may opt for specialities with higher pay or better job prospects (Aghukwa, 2010; Akpayak et al., 2014; Asani et al., 2016). Psychiatry is often perceived as a less lucrative and less prestigious speciality when compared to other areas, such as surgery, which may discourage students from pursuing it (Asani et al., 2016).

One of the most potent intra-medical school factors is quality exposure. In a 20-country cross-sectional study, Farooq et al. (2014) found that quality exposure to psychiatry education is significantly associated with a career choice in psychiatry. It also highlighted the value of participation in simulation activities and exposure to clinical settings to improve medical undergraduates’ attitudes toward the field of psychiatry. In contrast, didactic teaching appeared to have the opposite effect on recruitment. Another study found that these active learning activities were more effective at reducing mental illness stigma among medical students worldwide (Corrigan et al., 2012) and in Nigeria (Ighodaro et al., 2015).

Enrichment activities, such as research and special study modules, are typical activities that go beyond standard teaching and clinical placement. Enrichment activities like quality exposure to psychiatry education have also been shown in various studies to enhance medical students’ attitudes toward psychiatry (Halder et al., 2013; Lyons, 2013; Farooq et al., 2014). Multiple studies over the years have implicated personality factors. In a study conducted in Spain, high sensitivity and low (impulsive) self-control were found in choosing psychiatry as a career (Monleón Moscardó et al., 2001). Another study discovered higher levels of neuroticism and openness among Australian medical students who went on to study psychiatry (Maron et al., 2007). However, no specific study has been conducted in Nigeria to discover any relationship between personality and the choice of psychiatry. Moreover, mentorship and career guidance programs are other notable factors in Nigerian medical schools. According to a study conducted by Ossai et al. (2016) among medical schools in Southeastern Nigeria, there was no institutional career guidance for the students. This could also be the case at other medical schools in Nigeria, depriving medical students of the exposure and mentorship they need to make informed career decisions.

## Recommendations

A holistic approach is crucial to encouraging enthusiasm and overcoming challenges that hinder undergraduate medical students, students from other fields, and others from entering mental health professions. This comprehensive strategy requires cross-disciplinary and stakeholder collaboration.

All medical and healthcare undergraduate programs should include robust mental health education to achieve this. While psychiatry directly addresses mental illness, primary care, nursing, social work and other disciplines also encounter mental health patients. Providing mental health education across various domains equips healthcare practitioners with a holistic perspective and the necessary skills to manage mental health issues effectively.

Furthermore, comprehensive community-based mental health awareness programs should extend beyond medical circles and engage the wider public, particularly the youth. These initiatives, including public seminars, workshops and informational campaigns, can educate citizens about the importance of mental health and the diverse career opportunities within the field.

Transdisciplinary teamwork plays a pivotal role in holistic mental health care. Collaboration among psychiatrists, psychologists, social workers, counselors and other mental health providers enhances the quality of patient treatment. Recognizing that mental health involves biological, psychological and social components underscores the need for collaborative efforts.

Enhancing support systems is critical for mental healthcare professionals to sustain their well-being throughout their careers. Addressing burnout and compassion fatigue, which are prevalent challenges, requires implementing measures that promote wellness, build resilience and prevent attrition, ultimately retaining talent in the field.

Structured mentorship programs can be highly beneficial, connecting prospective mental health professionals with experienced practitioners from various fields. Seasoned mental health professionals serving as role models inspire younger generations and illuminate the diverse career pathways within mental healthcare.

Additionally, establishing and actively promoting scholarships tailored for students pursuing careers in mental health professions, such as psychiatry, is imperative. These scholarships aim to make medical education more accessible and appealing to a broader range of students.

Prioritizing diversity and inclusion within mental health professions is essential. A diverse workforce representing various cultures, ethnicities and social groups enhances mental healthcare by fostering cultural competence and encouraging individuals from different backgrounds to pursue careers in this vital field.

National advocacy is pivotal for driving changes in mental healthcare and education policies. Collaborative efforts among policymakers can create incentives to attract students and professionals to mental health, potentially including financial incentive programs.

Finally, research and innovation are crucial in advancing mental health treatment. Engaging students, undergraduates and aspiring professionals from diverse backgrounds in research projects enhances their understanding of the field and ignites their passion for mental health.

By embracing this comprehensive set of recommendations, we can inspire students and citizens to pursue careers in mental health professions. This holistic strategy aims to cultivate a diversified, talented and compassionate workforce capable of seamlessly addressing Nigeria’s and the world’s pressing mental health challenges.

## Conclusion

Given Nigeria’s pressing mental health issues, a holistic approach is essential. The scarcity of mental health professionals and the increasing burden of mental illnesses underscore the need for immediate action. Crucial stages include integrating comprehensive mental health education into all healthcare programs, fostering community awareness and fostering collaboration among diverse healthcare providers.

Mentoring, scholarships and advocacy for mental healthcare should be prioritized to encourage future professionals and increase accessibility. Embracing diversity and innovation while conducting substantive research is essential for addressing this pressing issue. Together, these recommendations aim to cultivate a skilled and compassionate workforce capable of tackling Nigeria’s mental health challenges.

## Data Availability

Data sharing does not apply to this article as no datasets were analyzed.
