# Supplementary material for: Mental health challenges in Nigeria: Bridging the gap between demand and resources
Source: Glob Ment Health (Camb). 2024 Feb 16;11:e29. doi: 10.1017/gmh.2024.19 (PMC10988134; doi:10.1017/gmh.2024.19)
Supplement: Peer review history

# Editor report: Recommendation: Mental health challenges in Nigeria: Bridging the gap between demand and resources — R0/PR2

**DOI:** 10.1017/gmh.2024.19.pr2

**Affiliations:** Primary Healthcare Sciences, University of Zimbabwe Faculty of Medicine, Zimbabwe

No accompanying comment.

# Editor report: Decision: Mental health challenges in Nigeria: Bridging the gap between demand and resources — R0/PR3

**DOI:** 10.1017/gmh.2024.19.pr3

**Affiliations:** London School of Hygiene & Tropical Medicine, United Kingdom of Great Britain and Northern Ireland

No accompanying comment.

# Editor report: Recommendation: Mental health challenges in Nigeria: Bridging the gap between demand and resources — R1/PR5

**DOI:** 10.1017/gmh.2024.19.pr5

**Affiliations:** Primary Healthcare Sciences, University of Zimbabwe Faculty of Medicine, Zimbabwe

Kindly address the minor comments suggested by the reviewers before the article can be unconditionally accepted for publication.

# Editor report: Decision: Mental health challenges in Nigeria: Bridging the gap between demand and resources — R1/PR6

**DOI:** 10.1017/gmh.2024.19.pr6

**Affiliations:** London School of Hygiene & Tropical Medicine, United Kingdom of Great Britain and Northern Ireland

No accompanying comment.

# Editor report: Recommendation: Mental health challenges in Nigeria: Bridging the gap between demand and resources — R2/PR8

**DOI:** 10.1017/gmh.2024.19.pr8

**Affiliations:** Primary Healthcare Sciences, University of Zimbabwe Faculty of Medicine, Zimbabwe

No accompanying comment.

# Editor report: Decision: Mental health challenges in Nigeria: Bridging the gap between demand and resources — R2/PR9

**DOI:** 10.1017/gmh.2024.19.pr9

**Affiliations:** London School of Hygiene & Tropical Medicine, United Kingdom of Great Britain and Northern Ireland

No accompanying comment.
